# Author Correction: 2019–2020 H1N1 clade A5a.1 viruses have better in vitro fitness compared with the co-circulating A5a.2 clade

**DOI:** 10.1038/s41598-023-45263-4

**Published:** 2023-10-23

**Authors:** Nicholas J. Swanson, Paula Marinho, Amanda Dziedzic, Anne Jedlicka, Hsuan Liu, Katherine Fenstermacher, Richard Rothman, Andrew Pekosz

**Affiliations:** 1 W. Harry Feinstone Department of Molecular Microbiology and Immunology, The Johns Hopkins Bloomberg School of Public Health, 615 North Wolfe Street, rm W2116, Baltimore, MD 21205 USA; 2 Department of Emergency Medicine, Johns Hopkins University School of Medicine, Baltimore, MD USA

Correction to: *Scientific Reports* 10.1038/s41598-023-37122-z, published online 23 June 2023

The Acknowledgements section in the original version of this Article was incomplete,

“This work was supported by National Institutes of Health (NIH) contracts N272201400007C and N7503021C00045 for the Johns Hopkins Centers of Excellence in Influenza Research and Research, NIH T32 AI007417, as well as the Richard Eliasberg Family Foundation. We acknowledge the Protein-Glycan Interaction Resource of the CFG and the National Center for Functional Glycomics (NCFG) at Beth Israel Deaconess Medical Center, Harvard Medical School (supporting grant R24GM137763) for glycan microarray studies. The authors thank the healthcare workers who enrolled and participated in the Johns Hopkins Center for Excellence in Influenza Research and Surveillance study. We are grateful for the efforts of the clinical coordination team at JHH who collected samples. We thank the laboratories of Sabra Klein, Kimberly Davis, Nicole Baumgarth, and Andrew Pekosz for discussion of data and future directions.”

now reads:

“This work was supported by National Institutes of Health (NIH) contracts N272201400007C and N7503021C00045 for the Johns Hopkins Centers of Excellence in Influenza Research and Research, NIH T32 AI007417, NIH U54 AG062333, as well as the Richard Eliasberg Family Foundation. We acknowledge the Protein-Glycan Interaction Resource of the CFG and the National Center for Functional Glycomics (NCFG) at Beth Israel Deaconess Medical Center, Harvard Medical School (supporting grant R24GM137763) for glycan microarray studies. The authors thank the healthcare workers who enrolled and participated in the Johns Hopkins Center for Excellence in Influenza Research and Surveillance study. We are grateful for the efforts of the clinical coordination team at JHH who collected samples. We thank the laboratories of Sabra Klein, Kimberly Davis, Nicole Baumgarth, and Andrew Pekosz for discussion of data and future directions."

The original Article has been corrected.

