# Supplementary material for: 2019–2020 H1N1 clade A5a.1 viruses have better in vitro fitness compared with the co-circulating A5a.2 clade
Source: Sci Rep. 2023 Jun 23;13:10223. doi: 10.1038/s41598-023-37122-z (PMC10290074; doi:10.1038/s41598-023-37122-z)
Supplement: Supplementary file 1 — Supplementary Table S1. [file 41598_2023_37122_MOESM1_ESM.docx]

| **Chart Number** | **Glycan ID** | **Glycan Structure** |
| --- | --- | --- |
| 1 | 230 | Neu5Aca2-6(Neu5Aca2-3)GalNAca-Sp8 |
| 2 | 238 | Neu5Aca2-6(Neu5Aca2-3Galb1-3)GalNAca-Sp8 |
| 3 | 239 | Neu5Aca2-6(Neu5Aca2-3Galb1-3)GalNAca-Sp14 |
| 4 | 316 | Neu5Aca2-3Galb1-4GlcNAcb1-2Mana1-6(Neu5Aca2-6Galb1-4GlcNAcb1-2Mana1-3)Manb1-4GlcNAcb1-4GlcNAcb-Sp12 |
| 5 | 480 | Galb1-4(Fuca1-3)GlcNAcb1-6(Neu5Aca2-6(Neu5Aca2-3Galb1-3)GlcNAcb1-3)Galb1-4Glc-Sp21 |
| 6 | 55 | Neu5Aca2-6Galb1-4GlcNAcb1-2Mana1-6(Neu5Aca2-6Galb1-4GlcNAcb1-2Mana1-3)Manb1-4GlcNAcb1-4GlcNAcb-Sp12 |
| 7 | 56 | Neu5Aca2-6Galb1-4GlcNAcb1-2Mana1-6(Neu5Aca2-6Galb1-4GlcNAcb1-2Man-a1-3)Manb1-4GlcNAcb1-4GlcNAcb-Sp21 |
| 8 | 57 | Neu5Aca2-6Galb1-4GlcNAcb1-2Mana1-6(Neu5Aca2-6Galb1-4GlcNAcb1-2Mana1-3)Manb1-4GlcNAcb1-4GlcNAcb-Sp24 |
| 9 | 124 | Gala1-6Glcb-Sp8 |
| 10 | 125 | Galb1-2Galb-Sp8 |
| 11 | 127 | Galb1-3GlcNAcb1-3Galb1-4(Fuca1-3)GlcNAcb-Sp0 |
| 12 | 259 | Neu5Aca2-6GalNAca-Sp8 |
| 13 | 260 | Neu5Aca2-6GalNAcb1-4GlcNAcb-Sp0 |
| 14 | 261 | Neu5Aca2-6Galb1-4(6S)GlcNAcb-Sp8 |
| 15 | 262 | Neu5Aca2-6Galb1-4GlcNAcb-Sp8 |
| 16 | 263 | Neu5Aca2-6Galb1-4GlcNAcb1-3Galb1-4(Fuca1-3)GlcNAcb1-3Galb1-4(Fuca1-3)GlcNAcb-Sp0 |
| 17 | 264 | Neu5Aca2-6Galb1-4GlcNAcb1-3Galb1-4GlcNAcb-Sp0 |
| 18 | 265 | Neu5Aca2-6Galb1-4Glcb-Sp0 |
| 19 | 266 | Neu5Aca2-6Galb1-4Glcb-Sp8 |
| 20 | 267 | Neu5Aca2-6Galb-Sp8 |
| 21 | 293 | Neu5Aca2-6Galb1-4GlcNAcb1-2Mana1-6(Galb1-4GlcNAcb1-2Mana1-3)Manb1-4GlcNAcb1-4GlcNAcb-Sp12 |
| 22 | 300 | Neu5Aca2-6Galb1-4GlcNAcb1-2Mana1-6(GlcNAcb1-2Mana1-3)Manb1-4GlcNAcb1-4GlcNAcb-Sp12 |
| 23 | 311 | Galb1-4GlcNAcb1-2Mana1-6(Neu5Aca2-6Galb1-4GlcNAcb1-2Mana1-3)Manb1-4GlcNAcb1-4GlcNAcb-Sp12 |
| 24 | 319 | Neu5Aca2-6Galb1-4GlcNAcb1-3Galb1-3GlcNAcb-Sp0 |
| 25 | 321 | Neu5Aca2-6Galb1-4GlcNAcb1-3Galb1-4GlcNAcb1-3Galb1-4GlcNAcb-Sp0 |
| 26 | 334 | Neu5Aca2-6Galb1-4GlcNAcb1-2Mana1-6(Mana1-3)Manb1-4GlcNAcb1-4GlcNAc-Sp12 |
| 27 | 335 | Mana1-6(Neu5Aca2-6Galb1-4GlcNAcb1-2Mana1-3)Manb1-4GlcNAcb1-4GlcNAc-Sp12 |
| 28 | 336 | Neu5Aca2-6Galb1-4GlcNAcb1-2Mana1-6Manb1-4GlcNAcb1-4GlcNAc-Sp12 |
| 29 | 337 | Neu5Aca2-6Galb1-4GlcNAcb1-2Mana1-3Manb1-4GlcNAcb1-4GlcNAc-Sp12 |
| 30 | 355 | Neu5Aca2-6GlcNAcb1-4GlcNAc-Sp21 |
| 31 | 356 | Neu5Aca2-6GlcNAcb1-4GlcNAcb1-4GlcNAc-Sp21 |
| 32 | 366 | Neu5Aca2-6Galb1-4GlcNAcb1-3GalNAc-Sp14 |
| 33 | 398 | Galb1-4GlcNAcb1-6(Neu5Aca2-6Galb1-3GlcNAcb1-3)Galb1-4Glc-Sp21 |
| 34 | 444 | Neu5Aca2-6Galb1-4GlcNAcb1-6(Fuca1-2Galb1-3GlcNAcb1-3)Galb1-4Glc-Sp21 |
| 35 | 451 | Neu5Aca2-6Galb1-4GlcNAcb1-2Mana1-6(GlcNAcb1-4)(Neu5Aca2-6Galb1-4GlcNAcb1-2Mana1-3)Manb1-4GlcNAcb1-4GlcNAcb-Sp21 |
| 36 | 452 | Neu5Aca2-6Galb1-4GlcNAcb1-4Mana1-6(GlcNAcb1-4)(Neu5Aca2-6Galb1-4GlcNAcb1-4(Neu5Aca2-6Galb1-4GlcNAcb1-2)Mana1-3)Manb1-4GlcNAcb1-4GlcNAcb-Sp21 |
| 37 | 453 | Neu5Aca2-6Galb1-4GlcNAcb1-6(Neu5Aca2-6Galb1-4GlcNAcb1-2)Mana1-6(GlcNAcb1-4)(Neu5Aca2-6Galb1-4GlcNAcb1-2Mana1-3)Manb1-4GlcNAcb1-4GlcNAcb-Sp21 |
| 38 | 454 | Neu5Aca2-6Galb1-4GlcNAcb1-6(Neu5Aca2-6Galb1-4GlcNAcb1-2)Mana1-6(GlcNAcb1-4)(Neu5Aca2-6Galb1-4GlcNAcb1-4(Neu5Aca2-6Galb1-4GlcNAcb1-2)Mana1-3)Manb1-4GlcNAcb1-4GlcNAcb-Sp21 |
| 39 | 464 | Neu5Aca2-6Galb1-4GlcNAcb1-6(Galb1-3GlcNAcb1-3)Galb1-4Glcb-Sp21 |
| 40 | 467 | Neu5Aca2-6Galb1-4GlcNAcb1-6GalNAca-Sp14 |
| 41 | 468 | Neu5Aca2-6Galb1-4 GlcNAcb1-6(Neu5Aca2-6Galb1-4GlcNAcb1-3)GalNAca-Sp14 |
| 42 | 469 | Neu5Aca2-6Galb1-4GlcNAcb1-2Mana1-6(Neu5Aca2-6Galb1-4GlcNAcb1-2Mana1-3)Manb1-4GlcNAcb1-4(Fuca1-6)GlcNAcb-Sp24 |
| 43 | 474 | Neu5Aca2-6Galb1-4GlcNAcb1-6(Fuca1-2Galb1-4(Fuca1-3)GlcNAcb1-3)Galb1-4Glc-Sp21 |
| 44 | 489 | Neu5Aca2-6GalNAcb1-4(6S)GlcNAcb-Sp8 |
| 45 | 507 | Neu5Aca2-6Galb1-4GlcNAcb1-2Man-Sp0 |
| 46 | 560 | Neu5Aca2-6Galb1-4GlcNAcb1-6(Galb1-3)GalNAca-Sp14 |
| 47 | 46 | Neu5Aca2-3(6S)Galb1-4GlcNAcb-Sp8 |
| 48 | 63 | Fuca1-2Galb1-3GalNAcb1-4(Neu5Aca2-3)Galb1-4Glcb-Sp0 |
| 49 | 64 | Fuca1-2Galb1-3GalNAcb1-4(Neu5Aca2-3)Galb1-4Glcb-Sp9 |
| 50 | 143 | Galb1-3GalNAcb1-4(Neu5Aca2-3)Galb1-4Glcb-Sp0 |
| 51 | 214 | Neu5Aca2-3Galb1-4GlcNAcb1-3Galb1-4(Fuca1-3)GlcNAcb-Sp0 |
| 52 | 219 | Neu5Aca2-3Galb1-3GalNAca-Sp8 |
| 53 | 220 | Neu5Aca2-3Galb1-3GalNAca-Sp14 |
| 54 | 221 | GalNAcb1-4(Neu5Aca2-8Neu5Aca2-8Neu5Aca2-8Neu5Aca2-3)Galb1-4Glcb-Sp0 |
| 55 | 222 | GalNAcb1-4(Neu5Aca2-8Neu5Aca2-8Neu5Aca2-3)Galb1-4Glcb-Sp0 |
| 56 | 223 | Neu5Aca2-8Neu5Aca2-8Neu5Aca2-3Galb1-4Glcb-Sp0 |
| 57 | 224 | GalNAcb1-4(Neu5Aca2-8Neu5Aca2-3)Galb1-4Glcb-Sp0 |
| 58 | 226 | GalNAcb1-4(Neu5Aca2-3)Galb1-4GlcNAcb-Sp0 |
| 59 | 227 | GalNAcb1-4(Neu5Aca2-3)Galb1-4GlcNAcb-Sp8 |
| 60 | 228 | GalNAcb1-4(Neu5Aca2-3)Galb1-4Glcb-Sp0 |
| 61 | 229 | Neu5Aca2-3Galb1-3GalNAcb1-4(Neu5Aca2-3)Galb1-4Glcb-Sp0 |
| 62 | 231 | Neu5Aca2-3GalNAca-Sp8 |
| 63 | 232 | Neu5Aca2-3GalNAcb1-4GlcNAcb-Sp0 |
| 64 | 233 | Neu5Aca2-3Galb1-3(6S)GlcNAc-Sp8 |
| 65 | 234 | Neu5Aca2-3Galb1-3(Fuca1-4)GlcNAcb-Sp8 |
| 66 | 235 | Neu5Aca2-3Galb1-3(Fuca1-4)GlcNAcb1-3Galb1-4(Fuca1-3)GlcNAcb-Sp0 |
| 67 | 236 | Neu5Aca2-3Galb1-4(Neu5Aca2-3Galb1-3)GlcNAcb-Sp8 |
| 68 | 237 | Neu5Aca2-3Galb1-3(6S)GalNAca-Sp8 |
| 69 | 240 | Neu5Aca2-3Galb-Sp8 |
| 70 | 241 | Neu5Aca2-3Galb1-3GalNAcb1-3Gala1-4Galb1-4Glcb-Sp0 |
| 71 | 242 | Neu5Aca2-3Galb1-3GlcNAcb1-3Galb1-4GlcNAcb-Sp0 |
| 72 | 244 | Neu5Aca2-3Galb1-3GlcNAcb-Sp0 |
| 73 | 245 | Neu5Aca2-3Galb1-4(6S)GlcNAcb-Sp8 |
| 74 | 246 | Neu5Aca2-3Galb1-4(Fuca1-3)(6S)GlcNAcb-Sp8 |
| 75 | 247 | Neu5Aca2-3Galb1-4(Fuca1-3)GlcNAcb1-3Galb1-4(Fuca1-3)GlcNAcb1-3Galb1-4(Fuca1-3)GlcNAcb-Sp0 |
| 76 | 248 | Neu5Aca2-3Galb1-4(Fuca1-3)GlcNAcb-Sp0 |
| 77 | 249 | Neu5Aca2-3Galb1-4(Fuca1-3)GlcNAcb-Sp8 |
| 78 | 250 | Neu5Aca2-3Galb1-4(Fuca1-3)GlcNAcb1-3Galb-Sp8 |
| 79 | 251 | Neu5Aca2-3Galb1-4(Fuca1-3)GlcNAcb1-3Galb1-4GlcNAcb-Sp8 |
| 80 | 252 | Neu5Aca2-3Galb1-4GlcNAcb1-3Galb1-4GlcNAcb1-3Galb1-4GlcNAcb-Sp0 |
| 81 | 253 | Neu5Aca2-3Galb1-4GlcNAcb-Sp0 |
| 82 | 254 | Neu5Aca2-3Galb1-4GlcNAcb-Sp8 |
| 83 | 255 | Neu5Aca2-3Galb1-4GlcNAcb1-3Galb1-4GlcNAcb-Sp0 |
| 84 | 257 | Neu5Aca2-3Galb1-4Glcb-Sp0 |
| 85 | 258 | Neu5Aca2-3Galb1-4Glcb-Sp8 |
| 86 | 269 | Neu5Aca2-8Neu5Aca2-3Galb1-4Glcb-Sp0 |
| 87 | 281 | Neu5Aca2-3Galb1-4GlcNAcb1-6(Galb1-3)GalNAca-Sp14 |
| 88 | 287 | Neu5Aca2-3Galb1-3GlcNAcb1-3Galb1-3GlcNAcb-Sp0 |
| 89 | 288 | Neu5Aca2-3Galb1-4GlcNAcb1-3Galb1-3GlcNAcb-Sp0 |
| 90 | 309 | Neu5Aca2-3Galb1-4GlcNAcb1-6(Neu5Aca2-3Galb1-3)GalNAca-Sp14 |
| 91 | 315 | Neu5Aca2-3Galb1-4GlcNAcb1-2Mana1-6(Neu5Aca2-3Galb1-4GlcNAcb1-2Mana1-3)Manb1-4GlcNAcb1-4GlcNAcb-Sp12 |
| 92 | 320 | Neu5Aca2-3Galb1-3(Fuca1-4)GlcNAcb1-3Galb1-3(Fuca1-4)GlcNAcb-Sp0 |
| 93 | 326 | Neu5Aca2-3Galb1-4(Fuca1-3)GlcNAcb1-6(Neu5Aca2-3Galb1-3)GalNAc-Sp14 |
| 94 | 365 | Neu5Aca2-3Galb1-4GlcNAcb1-3GalNAc-Sp14 |
| 95 | 367 | Neu5Aca2-3Galb1-4(Fuca1-3)GlcNAcb1-3GalNAca-Sp14 |
| 96 | 381 | GalNAcb1-4(Neu5Aca2-3)Galb1-4GlcNAcb1-3GalNAca-Sp14 |
| 97 | 387 | Neu5Aca2-3Galb1-3GlcNAcb1-3GalNAca-Sp14 |
| 98 | 399 | Galb1-3GalNAcb1-4(Neu5Aca2-8Neu5Aca2-3)Galb1-4Glcb-Sp0 |
| 99 | 400 | Neu5Aca2-3Galb1-3GalNAcb1-4(Neu5Aca2-8Neu5Aca2-3)Galb1-4Glcb-Sp0 |
| 100 | 429 | Neu5Aca2-3Galb1-4GlcNAcb1-3Galb-Sp8 |
| 101 | 440 | Neu5Aca2-8Neu5Aca2-3Galb1-3GalNAcb1-4(Neu5Aca2-8Neu5Aca2-3)Galb1-4Glcb-Sp0 |
| 102 | 447 | Neu5Aca2-3Galb1-4GlcNAcb1-2Mana1-6(GlcNAcb1-4)(Neu5Aca2-3Galb1-4GlcNAcb1-2Mana1-3)Manb1-4GlcNAcb1-4GlcNAcb-Sp21 |
| 103 | 448 | Neu5Aca2-3Galb1-4GlcNAcb1-4Mana1-6(GlcNAcb1-4)(Neu5Aca2-3Galb1-4GlcNAcb1-4(Neu5Aca2-3Galb1-4GlcNAcb1-2)Mana1-3)Manb1-4GlcNAcb1-4GlcNAcb-Sp21 |
| 104 | 449 | Neu5Aca2-3Galb1-4GlcNAcb1-6(Neu5Aca2-3Galb1-4GlcNAcb1-2)Mana1-6(GlcNAcb1-4)(Neu5Aca2-3Galb1-4GlcNAcb1-2Mana1-3)Manb1-4GlcNAcb1-4GlcNAcb-Sp21 |
| 105 | 450 | Neu5Aca2-3Galb1-4GlcNAcb1-6(Neu5Aca2-3Galb1-4GlcNAcb1-2)Mana1-6(GlcNAcb1-4)(Neu5Aca2-3Galb1-4GlcNAcb1-4(Neu5Aca2-3Galb1-4GlcNAcb1-2)Mana1-3)Manb1-4GlcNAcb1-4GlcNAcb-Sp21 |
| 106 | 459 | Neu5Aca2-3Galb1-4GlcNAcb1-6(Neu5Aca2-3Galb1-4GlcNAcb1-3)GalNAca-Sp14 |
| 107 | 465 | Neu5Aca2-3Galb1-4GlcNAcb1-2Mana-Sp0 |
| 108 | 366 | Neu5Aca2-6Galb1-4GlcNAcb1-3GalNAc-Sp14 |
| 109 | 370 | Galb1-3GalNAca1-3(Fuca1-2)Galb1-4GlcNAc-Sp0 |
| 110 | 473 | Neu5Aca2-3Galb1-3GlcNAcb1-2Mana1-6(GlcNAcb1-4)(Neu5Aca2-3Galb1-3GlcNAcb1-2Mana1-3)Manb1-4GlcNAcb1-4GlcNAc-Sp21 |
| 111 | 478 | Neu5Aca2-3Galb1-3GlcNAcb1-6GalNAca-Sp14 |
| 112 | 513 | Neu5Aca2-3Galb1-3GlcNAcb1-2Mana-Sp0 |
| 113 | 516 | Neu5Aca2-3Galb1-3GalNAcb1-4Galb1-4Glcb-Sp0 |
| 114 | 521 | Neu5Aca2-3Galb1-4(Fuca1-3)GlcNAcb1-2Mana-Sp0 |
| 115 | 535 | Neu5Gca2-8Neu5Aca2-3Galb1-4GlcNAc-Sp0 |
| 116 | 538 | Neu5Aca2-8Neu5Aca2-3Galb1-4GlcNAc-Sp0 |
| 117 | 561 | GlcNAcb1-6(Neu5Aca2-3Galb1-3)GalNAca-Sp14 |
| 118 | 562 | Galb1-3GalNAcb1-4(Neu5Aca2-8Neu5Aca2-8Neu5Aca2-3)Galb1-4Glcb-Sp21 |
| 119 | 9 | Neu5Aca-Sp8 |
| 120 | 10 | Neu5Aca-Sp11 |
| 121 | 11 | Neu5Acb-Sp8 |
| 122 | 136 | Neu5Acb2-6(Galb1-3)GalNAca-Sp8 |
| 123 | 225 | Neu5Aca2-8Neu5Aca2-8Neu5Aca-Sp8 |
| 124 | 268 | Neu5Aca2-8Neu5Aca-Sp8 |
| 125 | 271 | Neu5Acb2-6GalNAca-Sp8 |
| 126 | 272 | Neu5Acb2-6Galb1-4GlcNAcb-Sp8 |
| 127 | 273 | Neu5Gca2-3Galb1-3(Fuca1-4)GlcNAcb-Sp0 |
| 128 | 274 | Neu5Gca2-3Galb1-3GlcNAcb-Sp0 |
| 129 | 276 | Neu5Gca2-3Galb1-4GlcNAcb-Sp0 |
| 130 | 277 | Neu5Gca2-3Galb1-4Glcb-Sp0 |
| 131 | 278 | Neu5Gca2-6GalNAca-Sp0 |
| 132 | 279 | Neu5Gca2-6Galb1-4GlcNAcb-Sp0 |
| 133 | 280 | Neu5Gca-Sp8 |
| 134 | 312 | Neu5Aca2-8Neu5Aca2-8Neu5Acb-Sp8 |
| 135 | 313 | Neu5Gcb2-6Galb1-4GlcNAc-Sp8 |
| 136 | 533 | Neu5Gca2-8Neu5Gca2-3Galb1-4GlcNAc-Sp0 |
| 137 | 534 | Neu5Aca2-8Neu5Gca2-3Galb1-4GlcNAc-Sp0 |
| 138 | 536 | Neu5Gca2-8Neu5Gca2-3Galb1-4GlcNAcb1-3Galb1-4GlcNAc-Sp0 |
| 139 | 537 | Neu5Gca2-8Neu5Gca2-6Galb1-4GlcNAc-Sp0 |

Supplementary Table S1: List of all glycans included in Figure 4 charts.
